# Characteristics of medication errors with parenteral cytotoxic drugs

**DOI:** 10.1111/j.1365-2354.2012.01331.x

**Published:** 2012-09

**Authors:** A Fyhr, R Akselsson

**Affiliations:** Division of Ergonomics and Aerosol Technology, Lund UniversityLund, Sweden

**Keywords:** medication errors, cytotoxic drugs, chemotherapy

## Abstract

Errors involving cytotoxic drugs have the potential of being fatal and should therefore be prevented. The objective of this article is to identify the characteristics of medication errors involving parenteral cytotoxic drugs in Sweden. A total of 60 cases reported to the national error reporting systems from 1996 to 2008 were reviewed. Classification was made to identify cytotoxic drugs involved, type of error, where the error occurred, error detection mechanism, and consequences for the patient. The most commonly involved cytotoxic drugs were fluorouracil, carboplatin, cytarabine and doxorubicin. The platinum-containing drugs often caused serious consequences for the patients. The most common error type were too high doses (45%) followed by wrong drug (30%). Twenty-five of the medication errors (42%) occurred when doctors were prescribing. All of the preparations were delivered to the patient causing temporary or life-threatening harm. Another 25 of the medication errors (42%) started with preparation at the pharmacies. The remaining 10 medication errors (16%) were due to errors during preparation by nurses (5/60) and administration by nurses to the wrong patient (5/60). It is of utmost importance to minimise the potential for errors in the prescribing stage. The identification of drugs and patients should also be improved.

## INTRODUCTION

Cytotoxic drugs are highly beneficial medications, but they must be used carefully because of their high toxicity and narrow therapeutic index, which means that there is little difference between lethal and therapeutic doses. Medication errors (MEs) with these drugs are not rare (Erdlenbruch *et al*. 2002) and are potentially fatal (Phillips *et al*. 2001) and should therefore be prevented. A review of the literature on MEs in chemotherapy, their incidences and characteristics, has recently been presented by Schwappach and Wernli (2010). When the characteristics of these MEs are identified actions and strategies can be suggested to prevent them.

Parenteral cytotoxic drug treatments are administered on an inpatient or outpatient basis. A team consisting of doctors, pharmacists and nurses is responsible for the prescription, preparation, administration and monitoring of the treatment. About 50 different cytotoxic drugs, including monoclonal antibodies, are used for parenteral administration in Sweden today. These drugs are administered in a wide variety of cancer therapies, both for curative and palliative care, and they are used in the treatment of small children up to elderly people. They can be used as a single drug or in combinations in complex regimes over several consecutive days repeated after 2–3 weeks. For most of the drugs, the dose is based on body surface area or other patient-specific factors (e.g. weight, renal function). Most cytotoxic drugs have a narrow therapeutic index. At the same time, for some of these drugs, such as cytarabine, and methotrexate, dosages vary widely depending on the condition being treated, how the drug is used, and the use of supportive therapy. Cytotoxic drugs, given parenterally and orally, are classified as ‘high-alert medications’ according to the Institute for Safe Medication Practice, USA.

Sweden has about nine million citizens. There are 65 hospitals varying in size; seven of them are university hospitals. During 2008 about 42 000 people got a cancer diagnosis for the first time, of these 330 were children (<20 years) (Swedish Cancer Society 2010). Approximately 350 000 parenteral cytotoxic preparations are prepared annually (2008). Most of them, 330 000, are prepared by hospital pharmacists (legislation requires that pharmacists have at least a bachelor's degree for preparation of cytotoxic drugs), and the rest by nurses in the unit (D. Svedmyr, Apoteket Farmaci AB, February 2011, personal communication). At the time of the study hospital pharmacies were run by a governmental company, Apoteket AB, and thus by an external partner to health care.

Healthcare providers in Sweden are legally obliged to report serious injuries and risks of injuries to the National Board of Health and Welfare (NBHW) under the Act on Professional Activity in Health and Medical Services (lex Maria). The events are registered and thoroughly investigated by NBHW. They are collected in a national database for analysis, and feedback is provided to healthcare practitioners and the public. All hospitals and pharmacies have local incident reporting systems, most of them now computerised. The incidents reported by hospital staff are assessed by a person appointed by the management. If the incident is judged to be serious, it is sent to the medical director of the hospital who is responsible for the final decision to report or not according to lex Maria. For the pharmacies the final decision has been centralised to the quality department. In 2008 a total of 1102 incidents were reported according to lex Maria (National Board of Health and Welfare 2011). About 250 (23%) of these involved a medication (U. Fryksmark, The National Board of Health and Welfare, February 2011, personal communication). Reports according to lex Maria were, after investigation, reported to a national risk database, administered by the NBHW. Complaints filed to the Medical Responsibility Board (HSAN) were also reported to the national risk database. HSAN was a national authority which assessed medical negligence. If healthcare staff was at fault the Board could take disciplinary action against them, giving admonition or warning. Anyone who was or had been a patient could file a complaint to the Board. If the patient him/herself was incapable of filing the complaint, it may be filed by a close relative. The NBHW, the Parliamentary Ombudsman and the Chancellor of Justice could also file complaints to the Board. The role of HSAN to judge medical negligence ended in 2010 and the role of NBHW has changed.

The purpose of reporting incidents is to learn in order to make improvements. Reporting systems often fail to fulfil their intended role because they are underused (Erdlenbruch *et al*. 2002). Information on reported MEs should be shared with other institutions, so that many can learn from the errors of a few. It could thus be of value to present the experiences from Sweden and 13 years of case reports to the national regulators on MEs with parenteral cytotoxic drugs. The errors were analysed in detail in order to gain as much knowledge as possible from them. The definition of an ME used is the one proposed by Ferner and Aronson (2006): ‘A medication error is a failure in the treatment process that leads to, or has the potential to lead to, harm to the patient’.

The aim of this study was to identify the characteristics of the MEs involving parenteral cytotoxic drugs in Sweden in order to answer the following questions: Which drugs were involved? What types of errors were made? Where in the medication use process did the errors take place? How were these errors discovered? What were the consequences for the patients?

## MATERIALS AND METHODS

Cases reported to the national error reporting systems have been used for a retrospective qualitative analysis. The inclusion criteria for this study are: A ME reported according to the lex Maria Act or to the Medical Responsibility Board (HSAN) between 1996 and 2008 involving a cytotoxic drug (ATC classification L01) and administered parenterally at a hospital. Problems with blood tests or other necessary tests during the treatment period are included if they result in the wrong treatment. Misdiagnoses, subcutaneous drug extravasation of the infusion, or problems with peripheral or central venous line during administration are excluded. Several reports on the same case were counted as one ME.

The material consists of ME reports obtained in the following ways:

- Reports retrieved from the national risk database from 1996 to mid-2006. A total of 101 reports were found; of these 44 met the inclusion criteria. Most of the reports excluded involved oral cytotoxic drugs.
- Reports retrieved from the NBHW database as the result of a search for reports involving the word ‘cytostatika’ for 2006–2008. A total of 12 reports were found; of these eight met the inclusion criteria.
- Eight reports were found using other sources: in a report retrieved from the national risk database (1), a colleague informed from another hospital pharmacy (4), the incident occurred at the university hospital where one of the authors worked (3).

**Figure 1:**
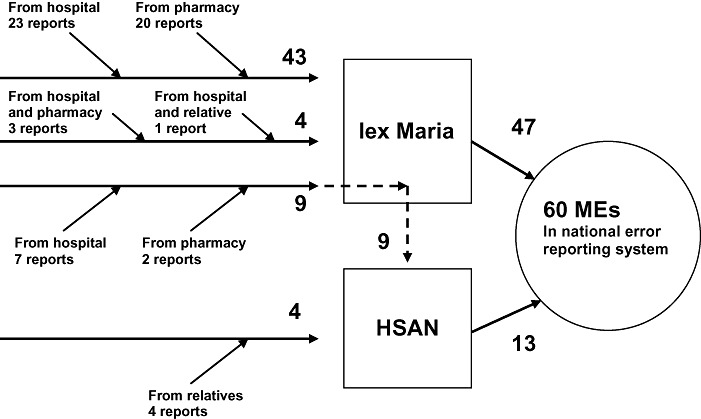
Origin of reports. A total of 56 reports were filed according to lex Maria. Nine of these were reported to HSAN from National Board of Health and Welfare, together with four reports from relatives a total of 13 were investigated by HSAN. HSAN, Medical Responsibility Board; ME, medication error.

The MEs were reported from the whole country and according to the content in Figure 1.

A total of 60 MEs meeting the inclusion criteria were found. The case reports were read and tables were compiled based on:

- Cytotoxic drugs involved.
- Type of error: wrong dose (too high, too low), wrong drug, wrong patient, wrong ambulatory pump, other.
- Where the error occurred in the medication use process (i.e. in prescribing and transcribing, preparation or administration).
- The error detection mechanisms (i.e. how and by whom the error was discovered).
- The consequences for the patient according to the NCC MERP Index for Categorising Medication Errors, USA (National Coordinating Council for Medication Error Reporting and Prevention 2011). This index was used for classification of the severity of the outcome: Category B–D *Error, No harm*; Category E–H *Error, Harm*, and Category I *Error, Death* (i.e. an error occurred that may have contributed to or resulted in the patient's death). Category A is *No Error* and thus was not included.

In 50 (83%) of the MEs the patient was an adult, and in 10 (17%) of the events a child.

## RESULTS

Table 1 gives examples of some representative MEs. A brief compilation of all 60 MEs can be found in Lund University Publications (2011).

**Table 1:** Examples of medication errors reported according to lex Maria and/or HSAN

ID no	Report	Drug	Where	What happened/*discovered/*consequences
#6	lex Maria	Cisplatin should have been cyclophosphamide	University hospital	Patient received another patient's drug, 30 mg of cisplatin instead of cyclophosphamide. *Nurse discovered during further preparation and informed the doctor.* The patient had to stay at hospital for 1 night. The treatment was delayed for 1 week. No permanent harm.
#16	lex Maria	Doxorubicin	Pharmacy	Pump run at too high a speed used during preparation; homepump delivered drug during 1 instead of 48 h. *Discovered by patient/nurse when the infusion was so quick.* Extra treatment prescribed. Probably no harm.
#18	lex Maria	Vincristine	University hospital	Dose that was 10 times higher than prescribed. A dose of 2.0 mg became 20 mg when prepared by a nurse. *Discovered the same afternoon during nursing rounds; her colleagues reacted.* Serious neurological harm; treated in respirator for a period. The patient died after 7 months.
	HSAN			
#19	lex Maria	Cisplatin	Pharmacy	Double dose prepared. Prescription ‘Cisplatin 0.5 mg, 190 mg, 380 mL to be diluted in 2 × 1000 mL NaCl 9 mg/mL’ was interpreted as a dose of 380 mg. *The first pharmacist pondered the dose in the evening, contacted the hospital and the error was discovered.* Patient became deaf.
	HSAN			
#40	HSAN	Etoposide	University hospital	Total dose for the course became dose per day, 330 mg, 3 times per day for 3 days, should have been 110 mg, 3 times per day for 3 days. *Nurse suspected that the dose was too high and treatment was not given on day 3*. Patient suffered from anaemia and was hospitalised for two weeks.
#53	lex Maria	Carboplatin	County hospital	Prescription for 5 days should have been only for 1 day. Due to hearing disturbances from cisplatin, there was a switch to carboplatin. Dose 800 mg per day. *Discovered when the patient came back with adverse reactions, hospitalised for a week.* Probably no long-term harm.

The most commonly involved cytotoxic drugs were fluorouracil, followed by carboplatin, cytarabine and doxorubicin (Table 2). In five of the cases two drugs were involved in the error. In three of the errors involving fluorouracil the wrong ambulatory pumps were used in preparation. This resulted in the dose being delivered too fast. Fluorouracil doses that were too high were prescribed and there were mix-ups with other drugs during preparation. Two of the errors involving carboplatin occurred when it replaced cisplatin due to the latter's adverse effects on the kidneys and hearing. Carboplatin should then have been given for only 1 day but was mistakenly given for 3 or 5 days, which is the normal length of the treatment for cisplatin. One child and two adult patients received overdoses of carboplatin due to a misinterpretation of the Calvert formula on two different occasions. There was also a mix-up during preparation by the nurse resulting in the use of carboplatin instead of cisplatin. Total dose for a treatment period was misinterpreted as dose per day leading to overdoses of carboplatin and melphalan. For cytarabine four of the errors were too high doses, including two cases with tenfold errors, both of which occurred during preparation at the pharmacy. Four of the errors involving doxorubicin were too high doses, a mix-up with epirubicin and the use of the wrong ambulatory pump.

**Table 2:** Cytotoxic drugs involved in the medication errors and consequences for the patients

Drug	Number of medication errors (including when used in combinations of drugs)	Category of medication error* (*n*)
Fluorouracil	9	Death (1); harm (3); no harm (5)
Carboplatin	6 (7)	Death (1); harm† (5); no harm (1)
Cytarabine	6 (7)	Harm (2); no harm (4)
Doxorubicin	4 (7)	No harm (4)
Vincristine	4 (6)	Harm (3); no harm (1)
Cisplatin	4	Death (1); harm (2); no harm (1)
Etoposide	4	Death (1); harm (2); no harm (1)
Cyclophosphamide	2 (3)	No harm (2)
Melphalan	2 (3)	Death (1); harm (1)
Methotrexate	2	Harm (1); no harm (1)
Others	12	Harm (4); no harm (8)
Doxorubicin and vincristine	2	Harm (1); no harm (1)
Carboplatin and melphalan	1	Death (1)
Daunorubicin and cytarabine	1	No harm (1)
Doxorubicin and cyclophosphamide	1	Harm (1)
Total	60	Death (6); harm (25); no harm (30)

* Note: Category I *Error, Death* is an error that may have contributed to or resulted in the patient's death.

† One medication error involved two patients.

Each of the MEs was classified into one of six categories (Table 3). Doses that were too high originating from prescribing and transcribing or preparation was the largest category. Tenfold errors were made by doctors (cyclophosphamide), pharmacists (cytarabine, twice) and nurses (vincristine). In two of the MEs, including the tenfold error with cyclophosphamide, the error occurred during transcription from the doctors' prescriptions to the orders to the pharmacy. These have to be signed by the doctor making him/her responsible for the error. The wrong drug being used during preparation, both by pharmacists and nurses, or prescription was the second largest category. Examples of mix-ups between drugs were vincristine–vinblastine, docetaxel–paclitaxel and cytarabine–ifosphamide. Totally, there were 18 cases where drugs were mixed up. The wrong ambulatory pump was used during preparation by pharmacists in four cases, typically resulting in too quick a rate of infusion. In five of the MEs the drug was administered to the wrong patient.

**Table 3:** Error type and where in the medication use process the error occurred

Error	Prescribing and transcribing by doctors	Preparation by pharmacist	Preparation by nurse	Administration by nurses
Wrong dose: too high	18	7	2	
Wrong drug	3	13	2	
Wrong patient				5
Wrong ambulatory pump		4		
Wrong dose: too low or not specified	1	1		
Other	3		1	
Totally	25	25	5	5

**Figure 2:**
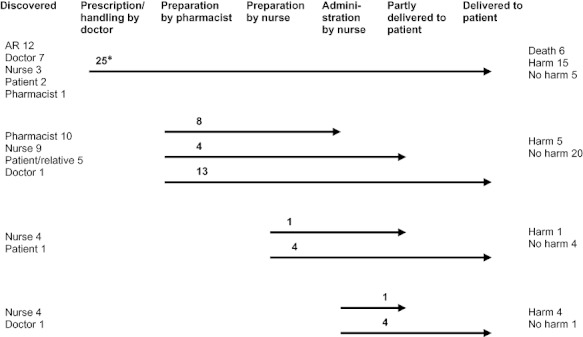
Start and fate of the investigated medication errors (MEs). It shows if the drugs were delivered to the patient or if the error was intercepted. The left column lists who discovered the ME or if it was discovered due to an adverse reaction (AR). The right column lists the consequences for the patients. *One ME involved two patients.

Twenty-five of the MEs (42%) occurred when doctors were prescribing or transcribing an order to the pharmacy. Another 25 of the MEs (42%) occurred within the pharmacies, and the remaining 10 MEs (16%) occurred when the nurses prepared (five MEs) or administered the drug to the wrong patient (five MEs) (see Figure 2). When the ME started at the prescribing stage, all of the cytotoxic preparations were delivered to the patient. The mistakes were revealed by an adverse reaction in the patient or found later by a professional or the patient. There were cases where the first dose in a treatment regimen was given and then the error was discovered. The doctor was informed and further treatment could be corrected or adjusted and necessary supportive care given.

If the ME started at the pharmacy, nurses stopped delivery of the infusion to the patient in eight of the cases. In Sweden nurses have to check the labelling from the pharmacy to see if it corresponds to the prescription written by the doctor. Five of the intercepted MEs were discovered this way. The others were discovered due to precipitations in one case, and to the wrong colour of the infusion in two cases (should have been yellow for methotrexate and red for doxorubicin). A pharmacist intercepted the ongoing treatment in four cases, and in 13 cases the drug was delivered to the patient. When the erroneous preparations were prepared by a nurse, all but one were delivered to the patient. It was the same for administration of a preparation to the wrong patient: all but one were delivered.

An accumulation of MEs can be seen on two occasions, reported from two different pharmacies. Five MEs that were reported in 1997 occurred within 6 months and five MEs that were reported in 2008 occurred within slightly over a month. In the first case the NBHW concluded that there were lack of routines, poor working conditions and inadequate organisation. The director was held responsible, and not the dispensing pharmacist. The second case was a transfer of preparations from another unit to the pharmacy in question, giving an increase in number of preparations with 35%. The NBHW criticised that no risk analyses had been performed before the transfer was decided.

The consequences for the patients were especially severe when the doctor made an error in prescribing and transcribing. Six of these MEs were judged as Category I, *Error, Death,* 15 as Category E–H, *Error, Harm,* and five as Category B–D, *Error, No harm*. When the ME started during preparation it led to *Harm* in five and *No harm* in 20 of the cases; 12 of them were intercepted.

## DISCUSSION

The most severe MEs in this study occurred during prescribing and transcribing by doctors. All six errors classified as *Error, Death* and 15 of the 25 errors classified as *Error, Harm* started at this stage. We are convinced that almost all MEs belonging to *Error, Death* were reported to the databases used in this study. Based on this we can state that most severe MEs in Sweden start with errors in prescribing and transcribing by doctors. Similar results were found in a study by Gandhi *et al*. (2005). In other studies the stage responsible for most of the errors (Rinke *et al*. 2007; Walsh *et al*. 2009) or the most fatal outcome was administration (Zernikow *et al*. 1999). The difference in results may be due to different material, to different definitions in the studies or to national/cultural differences.

It is interesting to note that of the 14 errors intercepted in total in our investigation, none started in the prescribing and transcribing stage. In a study from the USA, data from the U.S. Pharmacopeia Medication Errors Reporting Program was reviewed (Mehta *et al*. 1998). The authors found that in some of the cases (5/40) nurses and pharmacists intercepted the wrong medication which was ordered. Most certainly there are also such interceptions in Sweden, but for some reasons they are not reported according to lex Maria while some other interceptions of errors starting at other stages are. This points at an underreporting to the national database (lex Maria). The databases run by individual hospitals are hopefully better for learning from intercepted errors.

Nurses sometimes acted as barriers against errors occurring during preparation by pharmacists. Ways to improve the nurses' role as a barrier against errors ought to include thorough checking that the label and prescription correspond together with ample training and good experience. When nurses prepared or administered the drug to the patient, the error was seldom detected. In one of the cases the patient detected an error before the drug was administered. If patients are properly informed of the treatment, they can be involved in detection and prevention of errors as proposed in (Schwappach & Wernli 2010). Interestingly, in some cases both pharmacists and nurses realised their mistakes themselves the same day or within 24 h. This may be due to the practical handling of drug vials, syringes, infusions or patients. When they realised their mistakes they acted promptly to stop the infusion if it was possible and informed the doctor.

In several cases the drugs containing platinum caused serious consequences for the patients leading to death, hearing loss or depressed immune system and infection. Serious consequences with platinum-containing drugs are also described in (Mehta *et al*. 1998; Zernikow *et al*. 1999; Phillips *et al*. 2001). The same types of errors with the drugs found in this study have previously been presented in single case studies. This strengthens that we can learn from them and the precautions we can take in our organisations. In the single case studies, it was a misinterpretation of the Calvert formula that resulted in an overdose of carboplatin in two children (Liem *et al*. 2003). One case report describes a prescribing and administration error of cisplatin (Pourrat *et al*. 2004) and another report describes a cisplatin preparation error (Vila-Torres *et al*. 2009).

In this study the most common error types were wrong dose and wrong drug. This is similar to other studies, such as Mehta *et al*. (1998), Phillips *et al*. (2001) and Rinke *et al*. (2007). Only a few errors with too low dose (≤2 compared with 18 with too high doses) were reported in this study indicating underreporting. Too low dose can lead to therapeutic failure with serious consequences and should be reported and learnt from. Some of the causes for errors with too high doses or the wrong drug are well described. Tenfold or decimal point errors are a well-recognised risk to patients, existing in the prescribing, preparation and administration steps of the medication use system (Lesar 2002). Look-alike and sound-alike drugs in oncology may cause or contribute to potentially harmful MEs and there have been attempts to identify the drugs at risk (Schulmeister 2006; Kovacic & Chambers 2011). The problems with patient misidentification in oncology care have also been reported (Schulmeister 2008).

The pharmacies reported MEs where the error meant a risk for the patients; the drug was not fully delivered to the patient in nearly half of the MEs. The healthcare facilities reported only a few MEs where the administration was intercepted. This could be explained, for example, by differences in the judgement processes (lex Maria or not), by differences in organisations, size of organisations or culture. It may also be because pharmacies belong to another organisation and prefer to receive an independent judgement from the authorities.

In 10 (17%) of the cases the patient was a child. Compared with the number of children receiving cancer treatment every year this may be seen as high. There are several possible explanations: the great variation in size among children, dosing both according to body surface and per kg, and different methods for the calculation of body surface increasing the possibilities for errors. The treatment itself can be very complex and involve a risk to the patient. Another explanation may be that the parents closely follow the treatment and notice any problem, leading to more reports.

There are circumstances that have to be considered when interpreting this study. Cases from 1996 to 2008 were analysed and during this time there were several changes, such as in treatment protocols, drugs available, and in the work for improvements of patient safety. Furthermore, our data were limited to the content of the written reports from the NBHW or HSAN. The reports vary in quality and amount of information provided due to different authors and changes over the years. In the last years of the investigation period, the healthcare facility or pharmacy had made a root cause analysis before sending the report to NBHW, thus giving more comprehensive information. The data collected were limited to the content and ease of data extraction from the national risk database. Reporting to that risk database ended in mid-2006 making it difficult to find all relevant reports after that. In addition, due to one of the author's contacts with colleagues at pharmacies and with university hospital staff, there probably is an overrepresentation of MEs from these two venues. Another concern is underreporting. Thus, the number of MEs in the study period was probably not reflective of a true incidence rate. This may be supported by the fact that four of the 60 reports were filed by relatives of the patient and not by the healthcare facility. It is also supported, as already mentioned, by the fact that we had no MEs initiated at the prescribing and transcribing stage that were intercepted. Another problem is that there is no clear guidance on what no harm incidents to report. Thus the representativeness of incidents causing harm should be fairly good, but we cannot claim good representativeness for no harm incidents. In any case there are lessons to learn from the reported incidents and the lessons may be of value also for units with no or one incident. More reports and reports of good quality offer better opportunities for analysis and suggestions for improvements.

In this study we describe the characteristics of the MEs. Most of these error types identified have been described previously in the literature, which means that studies like this can be used as a source of information for improved patient safety. Eighteen high dose MEs occurred at the prescribing stage, but were not detected until administered to the patients. We could not find any patterns in types of hospitals in our material, but this kind of error should be followed to get better statistics. It is of utmost importance to minimise the potential for errors in the prescribing stage. This could be done using computerised physician (prescriber) order entry (CPOE) not only for prescribing but also for the whole medication use process. One example of development and implementation of CPOE is presented by Greenberg *et al*. (2006). The identification of drugs and patients should also be improved (e.g. by bar-coding). The use of bar-coding and telepharmacy during preparation has been presented by O'Neale *et al*. (2009). These technologies provide a means to improve the accuracy of preparations by decreasing the likelihood of using incorrect products or quantities of drug.

Prevention actions are taken. Since 2004 different CPOE systems have been introduced in Sweden. We estimate that almost 50% of all hospital units prescribing cytotoxic drugs use computerised system. Since 2007 nearly all pharmacies use a computerised system for their part of the process. A new project creating a national web-based encyclopaedia for cancer treatment, like Oncolex in Norway, has started and it will hopefully be in use early in 2013.

The next step will be to examine why these errors occurred. We plan to investigate the same material for system failures and missing barriers or barriers that did not capture the errors: Are there any common patterns? What other lessons can be learnt from these MEs and what countermeasures need to be taken?
